# Nucleolar Architecture Is Modulated by a Small Molecule, the Inositol Pyrophosphate 5-InsP_7_

**DOI:** 10.3390/biom13010153

**Published:** 2023-01-12

**Authors:** Soumyadip Sahu, Jacob Gordon, Chunfang Gu, Mack Sobhany, Dorothea Fiedler, Robin E. Stanley, Stephen B. Shears

**Affiliations:** 1 Inositol Signaling Group, Signal Transduction Laboratory, National Institute of Environmental Health Sciences, National Institutes of Health, Research Triangle Park, NC 27709, USA; 2 Nucleolar Integrity Group, Signal Transduction Laboratory, National Institute of Environmental Health Sciences, National Institutes of Health, Research Triangle Park, NC 27709, USA; 3 Cambridge Institute for Medical Research, Cambridge Biomedical Campus, Keith Peters Building, Hills Rd, Cambridge CB2 0XY, UK; 4 Department of Haematology, University of Cambridge School of Clinical Medicine, Jeffrey Cheah Biomedical Centre, Cambridge Biomedical Campus, Puddicombe Way, Cambridge CB2 0AW, UK; 5 Wellcome Trust-Medical Research Council Stem Cell Institute, Jeffrey Cheah Biomedical Centre, Cambridge Biomedical Campus, Puddicombe Way, Cambridge CB2 0AW, UK; 6 Leibniz-Forschungsinstitut für Molekulare Pharmakologie, Robert-Rössle-Str. 10, 13125 Berlin, Germany

**Keywords:** nucleolus, inositol, cell-signaling, ribosome, inositol pyrophosphate, phase separation, biomolecular condensate

## Abstract

Inositol pyrophosphates (PP-InsPs); are a functionally diverse family of eukaryotic molecules that deploy a highly-specialized array of phosphate groups as a combinatorial cell-signaling code. One reductive strategy to derive a molecular-level understanding of the many actions of PP-InsPs is to individually characterize the proteins that bind them. Here, we describe an alternate approach that seeks a single, collective rationalization for PP-InsP binding to an entire group of proteins, i.e., the multiple nucleolar proteins previously reported to bind 5-InsP_7_ (5-diphospho-inositol-1,2,3,4,6-pentakisphosphate). Quantitative confocal imaging of the outer nucleolar granular region revealed its expansion when cellular 5-InsP_7_ levels were elevated by either (a) reducing the 5-InsP_7_ metabolism by a CRISPR-based knockout (KO) of either *NUDT3* or *PPIP5K*s; or (b), the heterologous expression of wild-type inositol hexakisphosphate kinase, i.e., IP6K2; separate expression of a kinase-dead IP6K2 mutant did not affect granular volume. Conversely, the nucleolar granular region in *PPIP5K* KO cells shrank back to the wild-type volume upon attenuating 5-InsP_7_ synthesis using either a pan-IP6K inhibitor or the siRNA-induced knockdown of IP6K1+IP6K2. Significantly, the inner fibrillar volume of the nucleolus was unaffected by 5-InsP_7_. We posit that 5-InsP_7_ acts as an ‘electrostatic glue’ that binds together positively charged surfaces on separate proteins, overcoming mutual protein–protein electrostatic repulsion the latter phenomenon is a known requirement for the assembly of a non-membranous biomolecular condensate.

## 1. Introduction

**Figure 1:**
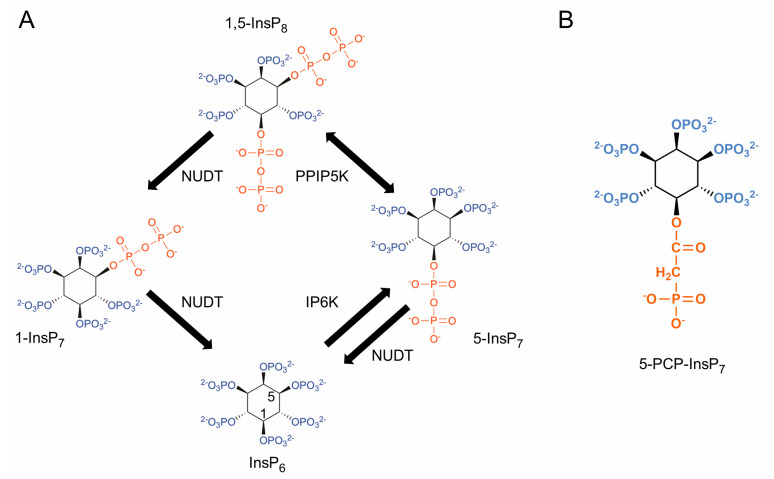
Structures of mammalian PP-InsPs and a metabolically resistant methylene bisphosphonate analog. (**A**) Structures of InsP_6_ and PP-InsPs. The graphic shows the proposed cyclical pathway for their interconversion in mammalian cells [3]. Mammalian cells express three isoforms of IP6Ks (types 1,2, and 3) and two PPIP5Ks (types 1 and 2). The latter each possess a kinase domain that phosphorylates 5-InsP_7_ to InsP_8_ and a separate phosphatase domain that dephosphorylates InsP_8_ back to 5-InsP_7_. NUDT3 (also known as DIPP1) is one of five mammalian NUDT isoforms in mammals that removes β-phosphates from both 5-InsP_7_ and InsP_8_. Note that little 1-InsP_7_ accumulates in HCT116 cells [4]. (**B**). Structure of metabolically resistant 5-PCP-InsP_7_.

Within the multitudinous cell-signaling family of inositol phosphates (InsPs), the inositol pyrophosphates (PP-InsPs; Figure 1A) comprise a specialized subgroup that is chemically, metabolically, and functionally distinct, all due to the presence of ‘energetic’ diphosphate groups [1,2,3]. The most well-studied of this group of polyvalent molecules are 5-diphosphoinositol-1,2,3,4,6-pentakisphosphate (5-InsP_7_) and 1,5-bis-diphosphoinositol-2,3,4,6-tetrakisphosphate (1,5-InsP_8_), which are synthesized by two key groups of enzymes: the inositol hexakisphosphate kinases (IP6Ks) and the diphosphoinositol pentakisphosphate kinases (PPIP5Ks) (Figure 1A). Considerable evidence indicates that these PP-InsPs are metabolically interconnected through a metabolic cycle [3], in which specialized phosphatases degrade 1,5-InsP_8_ through 1-InsP_7_ to InsP_6_; the most active of these phosphatases is DIPP1/NUDT3, which also dephosphorylates 5-InsP_7_ to InsP_6_ (Figure 1A).

Several biological activities have been attributed to the PP-InsPs. A few examples serve to illustrate this functional diversity: 1,5-InsP_8_ supervises the inorganic phosphate (Pi) balance in both plants [5] and animals [6]; this is metabolically critical not only because Pi is a fundamental component of the cell’s “energy currency” (i.e., ATP), but also because Pi is one of the most pervasive regulators of basic cellular metabolism [7]. Other studies have demonstrated that 1,5-InsP_8_ exerts control over cellular nucleotide synthesis, with sometimes dramatic consequences for cellular proliferation [8]. As for 5-InsP_7_, it regulates PolI-mediated rRNA transcription by *Saccharomyces cerevisiae* [9]. In mammals, 5-InsP_7_ regulates insulin secretion from pancreatic beta-cells [10,11], licenses high-fat diet-induced obesity [12], modulates the degradation of the plasma membrane sodium-potassium pump [13], and it inhibits mRNA decapping and promotes the accumulation of Processing (P-) bodies [14,15].

The molecular basis for some of the actions of PP-InsPs involves the modification of target protein functions by the donation of the molecules’ β-phosphoryl groups in a non-enzymic reaction known as protein pyrophosphorylation [16,17,18]. However, in part, due to the synthesis and application of metabolically resistant PP-InsPs such as 5-PCP-InsP_7_ (5-methylene-diphosphonate inositol 1,2,3,4,6-pentakisphosphate; Figure 1B), it is known that their non-covalent interactions with proteins expand the repertoire of PP-InsP signaling mechanisms [19]. Two examples of such mechanisms have emerged to date. First, the binding of PP-InsPs to certain PH domains can regulate the intracellular distribution of the host proteins [20,21,22]. The only other characterized PP-InsP-binding module is the SPX domain, but this is thought to be present in just one mammalian protein: XPR1 [6,23,24]. The latter is itself only known to have one function, namely, the regulation of the cellular efflux of Pi [6,23,25]. Consequently, it is thought that the rationalization of the functional diversity of this cell-signaling family depends upon the identification of other PP-InsP binding proteins; therefore there are ongoing efforts to catalog the entire PP-InsP interactome [19,26]. This approach has uncovered a bewilderingly large number of proteins that can associate with PP-InsPs, either directly or indirectly [19,26]. This situation is unprecedented for any other small-molecule intracellular signal, and its significance has not previously been rationalized.

Our goal has been to resolve this paradox in the current study. As an alternative to the reductive approach of attaching functional significance to the binding of a PP-InsP to a single protein, we have asked a question from a systems biology perspective: could there be a single, collective response to PP-InsP binding for an entire group of proteins? To find a potential answer, we have focused on the nucleolus, which contains a particularly high concentration of a variety of 5-InsP_7_-binding proteins, including components of RNA polymerase I complex, RNA polymerase III complex, and small nucleolar ribonucleoproteins [19,26,27]. Indeed, Arg- and Lys-rich sequences recur in nucleolar targeting motifs [28,29], thereby rendering all such proteins as candidates for ionic interactions with 5-InsP_7_, which has nine negative charges at a physiological pH. It has further been hypothesized that PP-InsPs can act as an ‘electrostatic glue’ that binds together positively charged surfaces on two separate proteins [2]. On a global scale, such a phenomenon could overcome protein–protein electrostatic repulsion and strongly elevate local protein concentrations, both of which are requirements for promoting the assembly of non-membranous biomolecular condensates [30]. Since the nucleolus is an important example of just such a phase-separated structure, we have hypothesized that the nucleolar volume might be increased by experimental procedures that elevate cellular 5-InsP_7_ levels. Herein we describe experiments that confirm that proposal.

## 2. Materials and Methods

### 2.1. Cell Culture

All strains of HCT116 cells were cultured in DMEM/F12 (Gibco catalog number: 11320033), according to the vendor’s (ATCC) recommendations. All cells were maintained in the media supplemented with 10% FBS (Gemini BenchMark™ FBS Catalog number: 100–106) and 1% antibiotic-antimycotic (Gibco catalog number: 15240062) in a cell culture incubator (37 °C, 5% CO_2_). These adherent cells were detached with 0.25% trypsin/EDTA (Gibco catalog number: 25200056) for passage. When required for the analysis of InsP_7_ and InsP_8_ levels (as described in [31]), cells were cultured in [^3^H]inositol as previously indicated [31]. The siRNA protocol for the simultaneous knockdown of IP6K1 and IP6K2 was exactly as previously described [14]. The TNP (N^2^-(m-trifluorobenzyl), N^6^-(p-nitrobenzyl)purine) treatment protocol was 10 µM added for 16 hr followed by an additional 10 µM for a further 2 h, as previously described [14].

### 2.2. Loading of 5-PCP-InsP_7_ into Cells Using Liposomes

Liposomes were prepared as 8 mg of lipid film inside a round-bottom glass flask, exactly as previously described [31]. As needed, the lipid film was hydrated (with vortexing) by the addition of 2 mL of 5 mM HEPES (pH 7.2 with NaOH) plus 0.25 µM 5-PCP-InsP_7_. A corresponding lipid film was prepared without the addition of 5-PCP-InsP_7_ (control liposomes). The liposomal dispersion was subjected to 5-10 freeze/thaw cycles: freezing at −80 °C for 30 min and thawing at 45 °C for 5 min. The liposomes were then sequentially extruded through two membrane filters, with pore sizes of 0.45 um and then 0.2 μm. The liposomes were stored in aliquots at 4 °C for up to 2 weeks. Cells (0.6 to 0.7 × 10^6^/well, plated on coverslips in 6-well MatTek dishes) were treated for 4 h with 2 µL liposomes in Pi-free DMEM plus 10% FBS.

### 2.3. Generation of NUDT3 KO Cells

HCT116 cells were grown at about 50% confluency (0.6 to 0.7 × 10^6^ cells/well) in a 6-well plate. *NUDT3* expression was disrupted using CRISPR-Cas9. HCT116 cells were transfected with 2 µg of 3 × sgRNA/Cas9 all-in-one expression clone targeting NUDT3 (Genecoepia catalog number: HCP291507-CG07-3), using 3 µL of the transfection reagent Lipofectamine-3000 (Invitrogen catalog number: L3000015) in 100 µL of Opti-MEM reduced serum medium (Gibco catalog number: 31985070). Next, single cells expressing GFP were sorted into 96-well plates 24 h post-transfection, and after cell line expansion, *NUDT3* KO clones were selected by Western analysis using a rabbit anti-NUDT3 antibody (Thermofisher catalog number: PA5-30435).

### 2.4. Lentiviral Transduction of HCT116 Cells

The pCDH-CMV-MCS-EF1a-CopGFP-T2A-Puro vector was purchased from System Biosciences (Catalog # CD513B-1). This vector was reengineered to form a pCDH-CMV-EF1a-MCS-P2A-CopGFP-T2A-Puro vector that enabled the insertion of genes under the control of the EF1a promoter. This vector was created by replacing the Xba I/Pst I fragment of the vector with a synthetic fragment from Genewiz Inc. that hosts EF1a-MCS-P2A-CopGFP. The full-length wild-type *IP6K2* or kinase-dead I*P6K2^K222A^* [32] was PCR amplified and cloned into the XhoI/BamHI site in the multiple cloning site of pCDH-CMV-EF1a-MCS-P2A-CopGFP-T2A-Puro.

All lentivirus were packaged and titered in HEK293T/17 cells (ATCC # CRL-11268) according to the published methods [33]. Briefly, 293T cells were transiently transfected with pMD2G, psPAX2, and a transfer vector containing the desired gene using Lipofectamine 2000. The supernatant was collected 48 h post-transfection and concentrated by centrifugation at 50,000× *g* for 2 h. Pellets were resuspended in PBS, aliquoted, and stored at −70 °C. All titers were determined by performing a digital droplet PCR to measure the number of lentiviral particles that integrated into the host genome. This titration was confirmed by using flow cytometry to monitor the co-expressed fluorescent moieties.

Lentiviral aliquots (MOI = 60) were added to HCT116 cells that had been plated at 0.1 × 10^6^ cells/well in a 6-well plate and cultured for 48 h, then were transferred into a virus-free medium for a further 48 h. Transduced cells were selected with puromycin (Thermofisher catalog number: A1113803, 2.5 µg/mL) and further confirmed by GFP fluorescence, i.e., images were obtained as 8x8 TILE SCANNING using a Zeiss Colibri epifluorescence microscope, and then further stitched using the Zeiss Zen STITCH function.

### 2.5. Western Blotting and Analysis

Cells were harvested and rinsed with ice-cold PBS; then, a protein extract was prepared using a RIPA Lysis and Extraction Buffer (ThermoFisher Scientific catalog number: 89901), supplemented with 1% (vol/vol) protease-phosphatase inhibitor cocktail (ThermoFisher Scientific catalog number: 78442). After quantification with a Pierce BCA protein assay kit (ThermoFisher Scientific catalog number: 23221), 25 µg of each protein extract was resolved by SDS-PAGE, transferred to a PVDF membrane, and probed with either rabbit anti-NUDT3 antibody (Thermofisher PA5-30435) or mouse anti-β-actin antibody (SantaCruz sc-47778, 1:5000). The secondary antibodies were either anti-rabbit IgG (Invitrogen 31460, 1:5000) or anti-mouse IgG (Cell Signaling Technology, 7076S, 1:5000). The immunoblots were developed using a SuperSignal™ West Pico PLUS Chemiluminescent Substrate (ThermoFisher Scientific catalog number: 34580) for HRP-conjugated secondary antibodies. All the blots were scanned using a Li-Cor Odyssey^®^ Fc imaging system and software (Li-Cor Biosciences). Densitometric analysis of the protein bands was performed using ImageJ (v 1.51j8).

### 2.6. Confocal Microscopy

Cells were grown on glass coverslips in 6 well dishes at approximately 50% confluency (0.6 to 0.7 × 10^6^ cells/well) in 2 mL DMEM/F-12 medium. Following experimental treatments, the medium was aspirated, and cells were washed with ice-cold PBS prior to fixation with 3.7% formaldehyde (Macron Chemicals catalog number: 5016) dissolved in PBS plus 1 mM CaCl_2_ and 0.5 mM MgCl_2_ (PBS+). Fixed cells were washed three times with PBS+/0.02% Triton X-100 followed by PBS+/0.1% Triton X-100). After the removal of the permeabilization solution, cells were incubated for 30 min with a blocking solution (PBS+/0.02% Triton X-100, 10% donkey serum (Sigma-Aldrich catalog number: D9663), 1% BSA (Fisher BioReagents catalog number: BP-9703) in). Then, the cells were incubated for 1 hr either with either a mouse anti-nucleostemin antibody (SantaCruz sc- sc-166460, 1:500) or a mouse anti-UBF1 antibody (SantaCruz sc- 13125, 1:100). For visualizing nucleostemin, the Alexa Fluor 488 donkey anti-mouse secondary antibody (Invitrogen A21202, 1:1200) was used. For visualizing UBF, the Alexa Fluor 594 donkey anti-mouse secondary antibody (Invitrogen A21203, 1:1200) was used. After further washing, the coverglasses were mounted on glass slides with ProLong^TM^ Glass Antifade with NucBlue^TM^ (Invitrogen catalog number: P36985) and sealed. Coverglasses were allowed to cure overnight prior to imaging using a Zeiss LSM780 confocal microscope (Carl Zeiss Inc, Oberkochen, Germany). Fluorescence images were captured using a 40X objective. Both the P-bodies and the nuclei were counted using the SPOT function in Imaris software (v9.5.0). The nucleolar signals were captured using the Z-stack function (5 × 1 µm Z-slices). The 3D rendering and volume measurements were performed using the SURFACE function in Imaris software [34,35].

### 2.7. Sucrose Density Gradient Fractionation of Ribosome Subunits

Cells were grown on two 150mm tissue culture dishes at approximately 80 % confluence. All growth media was then removed by aspiration, and the cells were rinsed with ice-cold PBS. After rinsing, PBS supplemented with cycloheximide at a concentration of 100 μg/mL was added to the dishes, which were then incubated at 37 °C for 15 min. The cells were harvested by scraping adherent cells from the plate and transferring them to a centrifuge tube which was then placed on the ice. Cells were spun down at 2000 rpm for 5 min. The cell pellet was then transferred to a 1.5 mL centrifuge tube. Cells were again pelleted, and all remaining PBS was then removed. The cell pellet was then resuspended in the Polysome Extraction Buffer (20 mM Tris-HCl pH 7.4, 60 mM KCl, 10 mM MgCl2, 1 mM DTT, 1% [v/v] Triton X-100, 0.1 mg/mL cycloheximide, 0.2 mg/mL heparin) supplemented with RNAse Inhibitor. Cells were nutated at 4 °C for one hour and then spun at 13,000 rpm at 4 °C for 30 min. RNA from cell lysate was quantitated using a Qubit fluorometer (Invitrogen) following the manufacturer’s protocol. A total of 150 ug of RNA was loaded onto a 7–47% sucrose gradient. Sucrose gradients were spun at 260,110× *g* at 4 °C for 2.5 h. The gradients were then fractionated using the Brandel BR-188 Density Gradient Fractionation System with a UV absorbance monitored at 254 nm.

### 2.8. Total RNA Analysis by Automated Electrophoresis

Cells were counted upon harvesting to ensure an equal number of cells per sample for the total RNA extraction as described above, and each sample was resuspended in 40 µl. RNA stocks were stored at −80 °C before use. Total ribosomal RNA was estimated by the quantification of the two largest mature rRNAs [36], 18S and 28S, in mammals, using a Bioanalyzer Automated Electrophoresis system (Agilent Technologies) with an RNA 6000 Nano Chip. Briefly, aliquots of the total RNA stocks were diluted with equal ratios to bring the sample concentration into the quantitative range of the Nano Chip (25—500 ng/uL). Nano Chip gel was prepared per instructions, stored at 4 °C, and used within 4 weeks of preparation. Gel-dye was added to the gel per instructions and immediately loaded into the Nano Chip before the loading sample. Diluted RNA samples were heat denatured at 70 °C for 2 min and immediately returned to ice before loading 1 uL of diluted sample into the Nano chip. Each sample was loaded on the chip in quadruplets for every biological sample; throughout the entire study, just one technical replicate exhibited a RIN number < 9.0, and it was discarded. The RNA assay data were manually imported into Excel for further analysis.

## 3. Results

### 3.1. Nucleolar Granular Volume Is Elevated in PPIP5K KO HCT116 Cells

It has previously been reported that the CRISPR-based knockout of the *PPIP5K*s interrupts the PP-InsP metabolic cycle (Figure 1A), leading to an elevation in 5-InsP_7_ levels in HCT116 cells; there is no off-target impact on the separate metabolic pathway to InsP_6_ synthesis [14,37]. Thus, we investigated the impact of the PPIP5K KO on nucleolar architecture. We first recorded the nucleolar granular volume in the intact cells. Previous studies have quantified the size of this nucleolar compartment by staining protein markers such as nucleostemin (NSM) and nucleophosmin [38,39,40]. The analysis of endogenously expressed proteins avoids potential artifacts that might arise from the heterologous expression of fluorescently tagged nucleolar proteins. We recruited endogenous NSM to record the nucleolar granular volume in wild-type and *PPIP5K* KO HCT116 cells.

**Figure 2:**
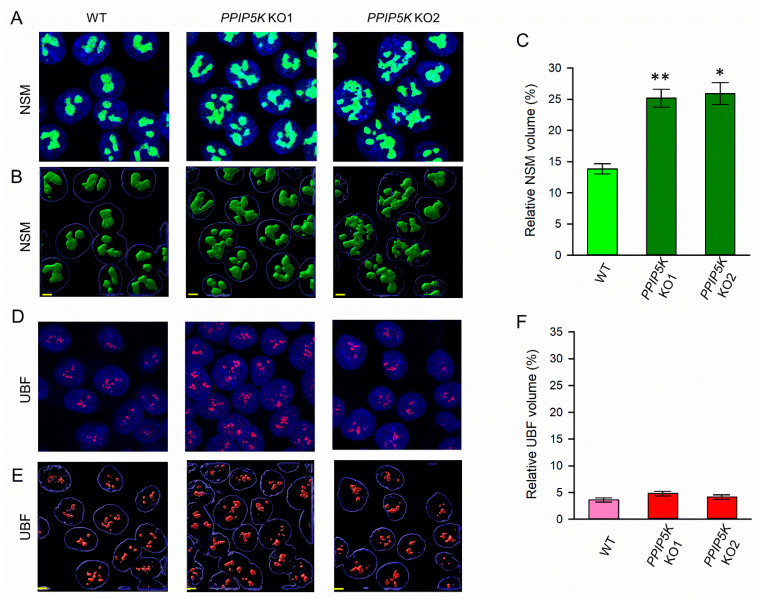
Nucleostemin (NSM) and UBF staining of wild-type and PPIP5K KO HCT116 cells. (**A**) Representative confocal image analysis for staining of nucleostemin (‘NSM’; colored green) in either wild-type (WT) cells or two independent clones of *PPIP5K* KO cells (KO1 and KO2). (**B**) Three-dimensional rendering of Z-stacks corresponding to the images in panel A. (**C**) Bar graphs represent means and standard errors from three independent Z-stack datasets (10 fields of view per condition, each containing at least six nuclei). (**D**)**,** Representative confocal image analysis for staining of UBF (colored red). (**E**) Three-dimensional rendering of Z-stacks corresponding to the images in panel D. (**F**) Bar graphs represent means and standard errors from three independent Z-stack datasets (10 fields of view per condition, each containing at least six nuclei). *, *p* < 0.05; **, *p* < 0.02. Scale bars (in yellow) depict 5 µm. The total nuclear volumes (µm^3^) were estimated from DAPI staining (blue) (means ± standard errors from 6 independent experiments): WT, 3543 ± 151; KO1, 3410 ± 176; KO2, 3457 ± 137.

To analyze the morphological consequences for the nucleolus in the *PPIP5K* KO cells, we combined confocal laser scanning microscopy with Airyscan to acquire two- and three-dimensional images, respectively (Figure 2A,B). In two independent clones of *PPIP5K* KO cells (KO1 and KO2), the total granular volume (as a ratio to the nuclear volume) was calculated to be 85% larger in both KO1 and KO2 lines (Figure 2C). By contrast, the DAPI-stained nuclear volume was very similar to that of the wild-type cells (see the legend in Figure 2).

### 3.2. The KO of PPIP5Ks Does Not Affect rRNA Synthesis

**Figure 3:**
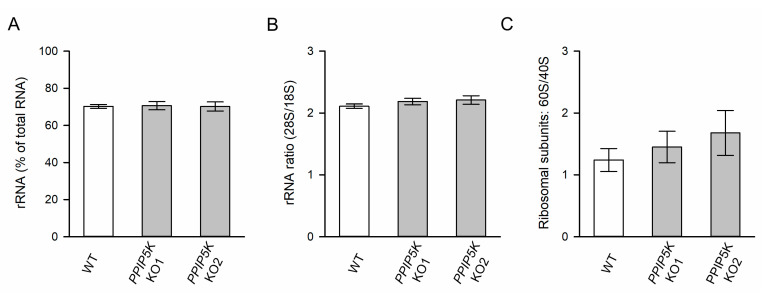
Analysis of ribosome production in wild-type and PPIP5K KO HCT116 cells. (**A**) Ribosomal RNA (rRNA) levels, (**B**) 28S/18S rRNA ratios, and (**C**) Ratios of 60S:40S ribosomal subunits, in wild-type HCT116 cells (open bars) and PPIP5K KO1 and KO2 HCT116 cells (gray bars), with standard errors, from five independent experiments. No statistically significant effects of the PPIP5K KO were observed (*p* > 0.05).

There are a considerable number of published studies that testify to an inexorable link between nucleolar morphology and ribosome biogenesis (e.g., [41]). The nucleolar fibrillar compartment is the site of rRNA transcription. The volume of this compartment was not affected by the *PPIP5K* KO, as measured by the staining of the RNA polymerase upstream binding factor, UBF (Figure 2D–F). Furthermore, the *PPIP5K* KO was not accompanied by any significant change in the cellular rRNA content, as determined by automated electrophoresis (Figure 3A). The *PPIP5K* KO also did not affect either 28S:18S rRNA ratios (Figure 3B) or 60S:40S ribosomal subunit ratios (Figure 3C). Thus, we conclude that elevated 5-InsP_7_ levels do not significantly alter ribosome biogenesis in HCT116 cells.

### 3.3. The Impact of NUDT3 KO upon Nucleolar Granular Volume

Mammalian cells express five isoforms of the NUDT/DIPP phosphatases (Figure 1A); we are not aware of any published studies with mammalian cells that have assayed the impact upon PP-IP levels for knocking out any of these phosphatases. However, kinetic studies with recombinant enzymes indicate that NUDT3/DIPP1 has the highest catalytic efficiency [42], and so we reasoned that cells in which the corresponding gene knocked out would show elevated levels of 5-InsP_7_, thereby yielding another method to study the impact of this PP-InsP upon the nucleolus.

**Figure 4:**
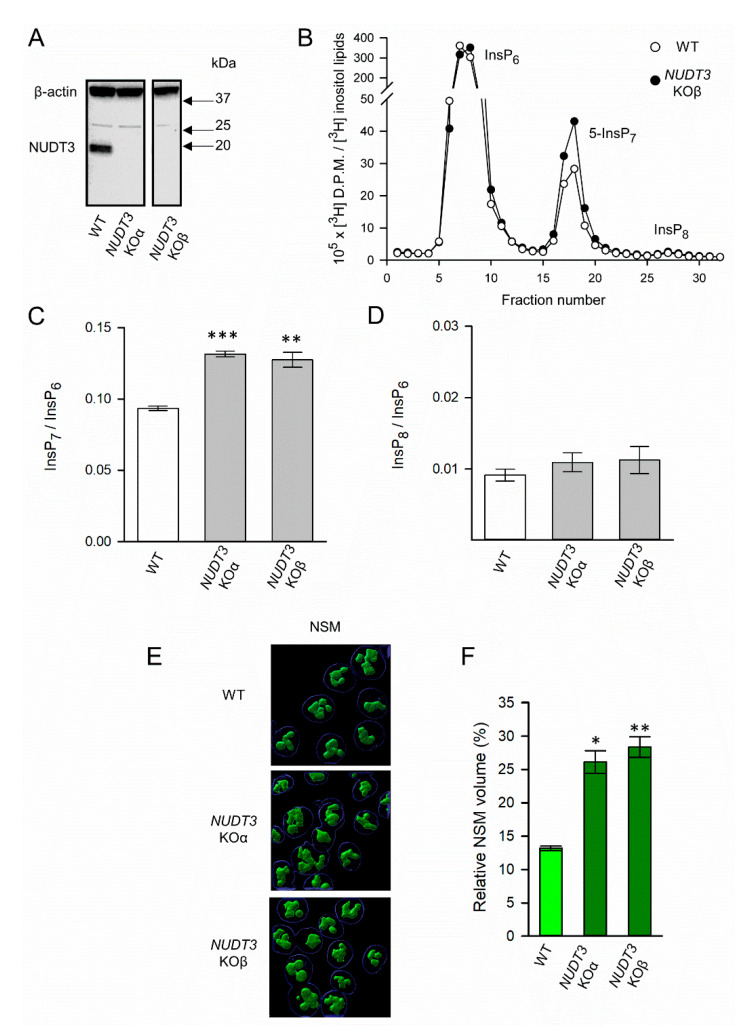
The effects of *NUDT3* KO upon PP-IP levels and NSM volume in HCT116 cells. (**A**) Western analysis of NUDT3 protein expression in WT HCT116 cells and two independent clones of *NUDT3* KO cells (KOα and KOβ). All lanes are from a single gel, from which non-relevant lanes have been removed. (**B**) HPLC resolution of [^3^H]-labeled InsP_6_, InsP_7,_ and InsP_8_ from [^3^H]-inositol radiolabeled wild type (open circles) and *NUDT3* KOβ (closed circles) HCT116 cells. (**C**) Cellular 5-InsP_7_ levels in WT (white bars) and *NUDT3* KOα and KOβ lines (gray bars). Data are means ± standard errors from four experiments. (**D**) Cellular 5-InsP_8_ levels in WT and *NUDT3* KOα and KOβ lines. Data are means ± standard errors from four experiments. (**E**) Representative three-dimensional rendering of Z-stack images of NSM volume in WT and *NUDT3* KOα and KOβ lines. (**F**) The bar graphs represent means and standard errors from three independent Z-stack datasets (10 fields of view per condition, each containing at least six nuclei). Scale bars (in yellow) depict five µm. *** *p* < 0.001, ** *p*< 0.01 * *p* < 0.02.

We have used CRISPR to create two independent clones of HCT116 *NUDT3* KO cells (namely KOα and KOβ; Figure 4A). The HPLC analysis of [^3^H]inositol-labeled cells showed that both clonal lines contained 40% higher levels of 5-InsP_7_ compared to WT cells (Figure 4B,C). This has no significant impact on InsP_6_, which is not surprising as its levels are approximately 20-fold higher than those of 5-InsP_7_ (Figure 4B). This is why we followed a usual practice in the field, which is to consider InsP_6_ as an internal standard to which levels of 5-InsP_7_ and InsP_8_ can be normalized. Significantly, in *NUDT3* KO cells, the nucleolar granular volume was about two-fold higher than that of wild-type cells (Figure 4E,F). This KO did not alter InsP_8_ levels (Figure 4B,D), which is consistent with the previous kinetic analysis of PPIP5Ks’ separate kinase and phosphatase activities that predict cellular InsP_8_ levels to be relatively insensitive to changes in 5-InsP_7_ concentration [43].

**Figure 5:**
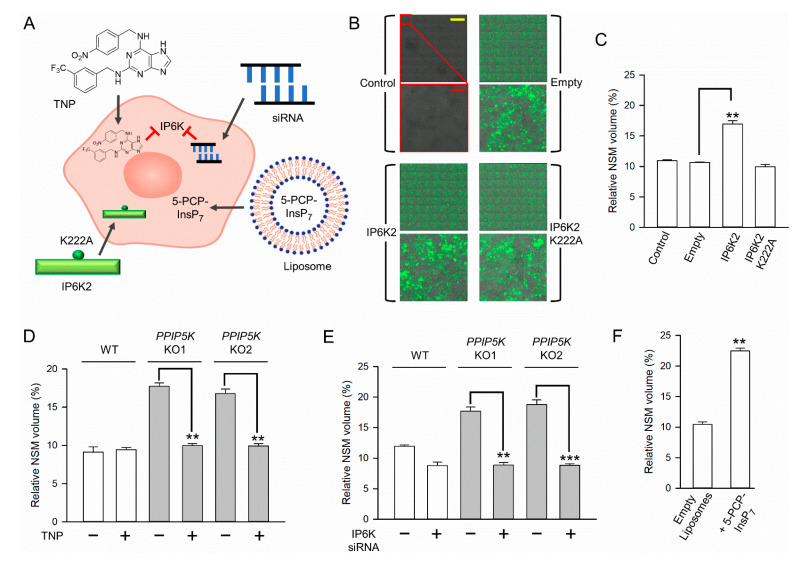

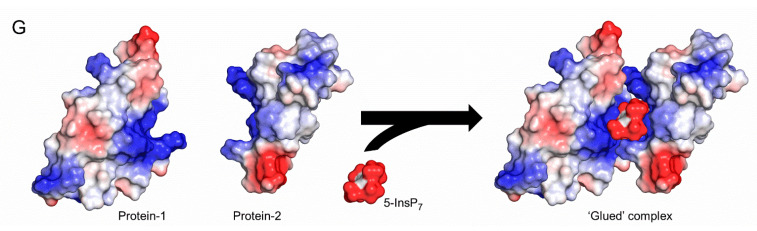
Direct pharmacological and molecular interventions in 5-InsP_7_ synthesis modify nucleolar granular volume in HCT116 cells. (**A**) Schematic to illustrate the various experimental approaches performed with HCT116 cells in this dataset (clockwise from top left), the pharmacological inhibition of IP6K by TNP; siRNA knockdown of IP6K1 + 2 by siRNA; liposomal delivery of non-hydrolyzable 5-PCP-InsP_7_; lentiviral-mediated stable expression of either wild-type IP6K2 or a Lys222Ala kinase-dead mutant (not shown in this graphic is the lentiviral co-expression of copGFP). (**B**) The copGFP fluorescence is shown in (clockwise from top left) non-transduced wild-type cells (‘Control’), or wild-type cells transduced with either lentiviral vector (‘Empty’), or vector hosting kinase-dead *IP6K2^K222A^*, or the vector hosting catalytically active *IP6K2*. The upper panels of each pair are stitched 20x fields of view; the top left corner of each upper panel (an individual field of view) is magnified 10-fold in each accompanying lower panel. Scale bars: yellow = 1 mm; red = 0.1 mm. (**C**) NSM volume in either non-transduced wild-type cells (‘Control’), or wild-type cells transduced with either lentiviral vector (‘Empty’), or vector hosting active *IP6K2*, or vector hosting kinase dead *IP6K2^K222A^*. (**D**) Effect of 10 µM TNP treatment (plus sign) or DMSO vehicle (minus sign) upon nucleolar volume in wild-type (white bars) or *PPIP5K* KO cells (gray bars). (**E**) The effect of siRNA against IP6K1/2 (noted with a plus sign) or corresponding controls (minus sign) upon NSM volume in wild-type (white bars) or *PPIP5K* KO cells (gray bars). (**F**) Effect upon NSM volume in wild-type cells after treatment with either empty liposomes or liposomes containing 5-PCP-InsP_7_. All data are mean values ± standard errors from three experiments. ** *p* < 0.01, *** *p* < 0.001. (**G**) A hypothetical electrostatic ‘glue’ to locally elevate protein concentration for promoting the formation of biomolecular condensates. Both 5-InsP_7_ (drawn as a space-filling model) and proteins-1 and -2 are depicted as electrostatic surface plots with the intensity of blue and red coloration denoting the degree of positive and negative electrostatic potentials at physiological pH. Any similarity to real protein structures is purely coincidental.

To further interrogate the relationship between cellular 5-InsP_7_ levels and nucleolar granular volume, we next performed a series of independent experiments in which we directly modulated the levels of this PP-IP by using several independent protocols (Figure 5A): the viral transduction of IP6K constructs, the pharmacological inhibition of IP6Ks, siRNA against IP6Ks, or delivery into cells of a metabolically stable 5-InsP_7_ analog using lipid nanoparticles. This application of multiple protocols represents a particularly robust approach to the identification of PP-InsP functionality.

### 3.4. The Impact upon Nucleolar Granular Volume of Changes in Cellular IP6K Activity

We increased the capacity of HCT116 cells to synthesize 5-InsP_7_ through the overexpression of IP6K2. Because IP6Ks have both catalytic and non-catalytic (scaffolding) roles, it is usual to compare the effects of wild-type enzymes versus a catalytically dead construct, along with fluorescent probes to monitor the expression [15]. Thus, in our experiments, we transduced HCT116 cells with lentivirus hosting either wild-type human IP6K2 or a catalytically dead IP6K2^K222A^ mutant [32] (Figure 5A,B). The co-expression of copGFP was tracked to the success of the transduction (Figure 5B). We found that the NSM volume was substantially elevated by wild-type IP6K2 but not by the kinase-dead mutant (Figure 5B,C), thereby demonstrating that the size of the nucleolar granular compartment correlates with the catalytic activity of this kinase (i.e., 5-InsP_7_ synthesis).

In separate experiments, we strongly reduced 5-InsP_7_ synthesis by treating wild-type and *PPIP5K* HCT116 KO cells for 18 hr with 10 µM of the pan-IP6K inhibitor, TNP, exactly as previously described for these cell types [14,31]. This pharmacological approach completely reversed the increase in the NSM volume that is normally observed in *PPIP5K* KO cells (Figure 5D). Another important aspect of this particular experiment is that *PPIP5K* KO cells cannot synthesize 1-InsP_7_ nor InsP_8_ [31]. Thus, the impact of TNP upon the nucleolar volume in these cells must be independent of these two PP-InsPs. We also decreased IP6K activity in HCT116 cells by the siRNA-mediated knockdown of IP6K1 plus IP6K2 exactly as previously described for these cells [14]. This IP6K1/IP6K2 knockdown in *PPIP5K* KO cells shrank the NSM volume to that observed in wild-type cells (Figure 5E).

### 3.5. Nucleolar Granular Volume Is Expanded by Liposomal Delivery of Metabolically Stable 5-PCP-InsP_7_

We recently described a procedure by which PP-InsPs could be encapsulated into liposomes for delivery into the cytoplasm of intact HCT116 cells [6,14]. It has been determined that liposomes will release their cargo into the cytoplasm following their endocytosis and delivery into tubular recycling endosomes [44]. We used this same procedure (Figure 5A) to deliver metabolically stable 5-PCP-InsP_7_ [45] into wild-type HCT116 cells. In these experiments, nucleolar granular volume was elevated approximately two-fold (Figure 5F). This is a particularly significant result because five-PCP-InsP_7_ cannot support protein pyrophosphorylation, which is one molecular mechanism by which PP-IPs can modify protein function [16,18]. Thus, we conclude that 5-InsP_7_ may regulate nucleolar volume through non-covalent interactions with appropriate target proteins [46].

## 4. Discussion

The main accomplishment of the current study has been to demonstrate that the granular portion of the nucleolus responds to multiple orthogonal, chemical, and genetic procedures, which all have in common the ability to manipulate 5-InsP_7_ levels in intact cells. For example, we show that the granular, NSM-positive volume expands in response to elevations in the 5-InsP_7_ accumulation brought about by the CRISPR-based KO of either *NUDT3* or *PPIP5K*s (Figure 2 and Figure 4); through the heterologous expression of wild-type IP6K2 (but not through the expression of the kinase-dead mutant, Figure 5B,C) the delivery into cells of 5-PCP-InsP_7_ was achieved (Figure 5F). Conversely, the nucleolar volume can be returned to the level seen in wild-type cells upon treatment with *PPIP5K* KO cells with either the pan-IP6K inhibitor TNP (Figure 5D), or by siRNA-induced knockdown of IP6K1+IP6K2 (Figure 5E). Our observation that 5-InsP_7_ influences the granular but not the fibrillar volume speak to the specificity of the action, which could be explained by functional interactions of this PP-InsP primarily with granular proteins.

This is an unprecedented demonstration that nucleolar architecture is modulated by a naturally occurring messenger molecule. Proteins and other macromolecules have been the focus of previous studies into electrostatic interactions that contribute to the dynamic regulation of the assembly, function, and disassembly of biomolecular condensates [47,48]. However, the 5-InsP_7_ molecule has an exceptional negative charge density and has previously been demonstrated to associate electrostatically with multiple nucleolar proteins [19,26,27]. Indeed, 5-InsP_7_ may act as an ‘electrostatic glue’ that binds together positively charged surfaces on two separate proteins (Figure 5G and [2]). At a nucleolar level, such a phenomenon could overcome general protein–protein electrostatic repulsion and strongly elevate local protein concentrations, both of which are requirements for promoting the assembly of non-membranous biomolecular condensates [30]. It is this process, we propose, that can explain how an increase in 5-InsP_7_ levels facilitates the expansion of the nucleolar granular compartment. This hypothesis takes PP-InsP research into a new direction by its emphasis on the collective significance of multiple ligand–protein interactions, as opposed to prior studies that attach separate biological outcomes of 5-InsP_7_ binding to individual proteins. This electrostatic model could, in theory, also apply to the other two mammalian PP-InsPs, 1-InsP_7,_ and 1,5-InsP_8_ (Figure 1). However, we deem this as an unlikely scenario, as these molecules are 10- and 50-fold less abundant than 5-InsP_7_ [3]. Moreover, both 1-InsP_7_ and 1,5-InsP_8_ cannot be synthesized by our *PPIP5K* KO cell line, which is used in the experiments described in Figure 5. We also do not exclude the possibility that the unique stereochemistry of 5-InsP_7_ may contribute to its regulation of nucleolar morphology.

The further biological significance of our study arises from it providing a new perspective on the impact of dynamic changes in the nucleolar volume, which have previously largely focused on the consequences for rRNA synthesis and ribosome biogenesis [41,47,49]. The rRNA is transcribed in the nucleolar fibrillar compartment; its volume was not affected by changes in the 5-InsP_7_ levels (Figure 2D,E,F). Moreover, we have shown that neither rRNA synthesis nor ribosome biogenesis is affected by elevations in the levels of 5-InsP_7_ (Figure 3).

In previously reported experiments with the yeast *S. cerevisiae* [9], rRNA synthesis was impaired upon the elimination of all IP6K activity following the deletion of the Kcs1 gene. The latter study concluded that the *Kcs1*Δ yeast strain exhibited a defect in transcriptional elongation due to the loss of the 5-InsP_7_-mediated pyrophosphorylation of PolI [9]. We cannot exclude the possibility that this PP-InsP may have a similar, constitutive role that maintains rRNA synthesis in mammals, because. In our study we did not completely eliminate 5-InsP_7_ synthesis from HCT116 cells. In any case, the mechanisms by which an elevation in 5-InsP_7_ levels regulates the nucleolar volume in the current study must involve a separate process that is independent of protein pyrophosphorylation, since it is recapitulated by the metabolically stable analog, 5-PCP-InsP_7_ (Figure 5E).

There are indications that our findings may have human health relevance. For example, a non-canonical function for the granular compartment of the nucleolus is homeostatic responses to certain cellular stresses (e,g. heat shock) [40]. Separately, it would appear to be detrimental for cells to exhibit sustained elevations in their nucleolar volume, which is inversely correlated with longevity [49]. Indeed, nucleolar size trends are smaller in humans if they adopt a healthier lifestyle and calorific reduction [49]. Pathological interest in the nucleolus is also concerned with neurodegeneration-related cellular events and cancer-related pathways [28]. Significantly, aging is associated with both an elevated nucleolar volume [47,49] and higher steady-state levels of 5-InsP_7_ [50]; these two phenomena are directly linked in the current study.

## Data Availability

The data that support the findings of this study are all provided within the body of the manuscript.
